# Systematic development of a text-driven and a video-driven web-based computer-tailored obesity prevention intervention

**DOI:** 10.1186/1471-2458-13-978

**Published:** 2013-10-20

**Authors:** Michel Jean Louis Walthouwer, Anke Oenema, Katja Soetens, Lilian Lechner, Hein De Vries

**Affiliations:** 1 School for Public Health and Primary Care (CAPHRI), Maastricht University, Maastricht, The Netherlands; 2 Department of Health Promotion, Maastricht University, P.O. Box 616, Maastricht 6200MD, The Netherlands; 3 Department of Psychology, Open University of the Netherlands, P.O. Box 2960, Heerlen 6419AT, The Netherlands

**Keywords:** Obesity, Physical activity, Diet, Intervention mapping protocol, Computer-tailoring, Weight management, Education level

## Abstract

**Background:**

This paper describes the systematic development of a text-driven and a video-driven web-based computer-tailored intervention aimed to prevent obesity among normal weight and overweight adults. We hypothesize that the video-driven intervention will be more effective and appealing for individuals with a low level of education.

**Methods and Design:**

The Intervention Mapping protocol was used to develop the interventions, which have exactly the same educational content but differ in the format in which the information is delivered. One intervention is fully text-based, while in the other intervention in addition to text-based feedback, the core messages are provided by means of videos. The aim of the interventions is to prevent weight gain or achieve modest weight loss by making small changes in dietary intake or physical activity. The content of the interventions is based on the I-Change Model and self-regulation theories and includes behavior change methods such as consciousness raising, tailored feedback on behavior and cognitions, goal setting, action and coping planning, and evaluation of goal pursuit. The interventions consist of six sessions. In the first two sessions, participants will set weight and behavioral change goals and form plans for specific actions to achieve the desired goals. In the remaining four sessions, participants’ will evaluate their progress toward achievement of the behavioral and weight goals. They will also receive personalized feedback on how to deal with difficulties they may encounter, including the opportunity to make coping plans and the possibility to learn from experiences of others. The efficacy and appreciation of the interventions will be examined by means of a three-group randomized controlled trial using a waiting list control group. Measurements will take place at baseline and six and twelve months after baseline. Primary outcome measures are body mass index, physical activity, and dietary intake.

**Discussion:**

The present paper provides insight into how web-based computer-tailored obesity prevention interventions consisting of self-regulation concepts and text-driven and video-driven messages can be developed systematically. The evaluation of the interventions will provide insight into their efficacy and will result in recommendations for future web-based computer-tailored interventions and the additional value of using video tailoring.

**Trial registration:**

NTR3501.

## Background

Obesity is a major global health problem [1,2]. The World Health Organization estimated that in 2008 about 502 million adults worldwide were obese [2] and it is expected that this figure will continue to rise in the future [1]. An individual is classified as obese when the Body Mass Index (BMI: weight in kilograms / height in meters^2^) is 30 or higher [3]. In the Netherlands, about 12% of the adult population is obese and it is expected that this proportion will rise to 18% by 2024 [4]. There is, however, a significant difference in obesity prevalence between individuals with a lower and a higher level of education [5-7]. In the Netherlands, 18.4% of individuals with a low level of education are obese, while this percentage is only 6.5% among people with a higher level of education, making the lower educated group an important target group for obesity prevention programs [5]. These high and increasing prevalence rates are worrying, particularly because obesity is associated with a range of health problems, such as type 2 diabetes, cardiovascular diseases, musculoskeletal disorders, psychological disturbance, different types of cancer, and premature death [8,9]. Obesity also causes a considerable economic burden, due to health care use, absenteeism, disability payments, and loss of productivity [1,8-10]. Because of the high burden of obesity, its prevention has become a high priority in many international and national public health policies [4,11].

Nevertheless, effective and efficient interventions to prevent obesity that can reach large numbers of people are scarce [12,13]. Moreover, these interventions are not always specifically geared to meet the needs of individuals with a low level of education [5,6,14]. Overall, it can be concluded that there is an urgent need for effective interventions to prevent obesity that can reach large numbers of people and that also appeal to and are effective for individuals with a lower level of education.

Ideally, obesity prevention interventions should have the possibility to adapt to an individual’s characteristics, because in general each individual wants to manage his/her body weight in his/her own specific way [15,16]. Web-based computer-tailored interventions may fulfill this requirement since these interventions can provide personally adapted messages based on an individual’s responses to, for example, an online questionnaire [17,18]. In the last decade, attention to web-based computer-tailored interventions has increased because of their potential advantages such as the ability to reach many individuals in a relatively cheap way [19] and the possibility for participants to use the intervention whenever they prefer [20]. Further, as web-based computer-tailored interventions require minimal human contact, they are also potentially cost-effective [21]. In addition, because the content of a web-based computer-tailored intervention can be adapted to an individual’s characteristics, it is suggested that messages are better read, saved, and remembered as compared to non-tailored materials [22]. Several reviews have already reported the promising effects of web-based computer-tailored interventions for modifying dietary and physical activity behavior [18,23] and preventing weight gain [24]. Web-based computer-tailored interventions may, therefore, be a good innovation to help in targeting the public health problem of obesity.

We developed two versions of a web-based computer-tailored intervention aimed to prevent obesity among Dutch adults with a healthy weight or limited overweight (BMI between 18.5 and 30). Both interventions have exactly the same educational content, but differ in the format in which the information is delivered. One intervention is fully text-based, without the use of visual elements (text-text intervention), while the other provides the core messages by means of videos with additional text-based information (video-text intervention). Although tailoring is considered to be an effective health education technique, the way messages are communicated may be even more important. We hypothesize that the video-text intervention will be more appealing and effective for individuals with a low level of education. Lower educated individuals have, for example, lower literacy skills, making textual messages less effective for these individuals [25]. Providing messages by means of other communication formats, such as videos, may therefore be a better way to reach these individuals [26]. An advantage of video messages is the fact that these do not require abstract text to be translated into concrete actions [27], which lower educated individuals often find harder to do [25,28]. Video messages reduce the cognitive effort needed to process information, which will free resources to process the main message and accordingly lead to a better understanding [29]. Research has already shown that videos can effectively attract attention and stimulate comprehension in lower educated individuals [26,30,31], while another study has shown a media preference in these individuals for video over text [32]. In addition, there is evidence that videos can be effective in improving cognitions such knowledge, attitude, self-efficacy, and intention [33,34] as well as actual behavior [35,36]. Hence, video-text interventions may be a promising approach to effectively increase the attention and comprehension of individuals with a low level of education, which will facilitate information processing and ultimately behavior change [37,38].

As the use of a planned approach during intervention development increases the likelihood for an intervention to be effective [39], we used the Intervention Mapping (IM) protocol [40] to develop the two versions of the intervention and the corresponding implementation and evaluation plan. The IM protocol is a framework for effective theory- and evidence-based decision making at each step in intervention development, implementation, and evaluation. The protocol consists of six succeeding steps that can be worked through in an iterative manner. The aim of this paper is to describe the systematic development of the two web-based computer-tailored interventions according to the six steps of the IM protocol.

## Methods and design

The interventions were developed by systematically going through the six steps of the IM protocol. In the first step of this protocol, a needs assessment is carried out using the PRECEDE model [41]. The second step provides the foundation for the intervention by specifying in detail who and what will be changed through the intervention. In step three, theory-based methods and practical applications are identified that can be used to achieve the intervention goals. Step four concerns the actual program development, by using all the products of the previous steps; during this step, a pretest of the intervention is conducted as well. In step five, adoption and implementation plans are specified. Finally, in step six, both an effect and process evaluation plan are developed.

### Step 1: needs assessment

Obesity is caused by an energy imbalance, in which energy intake exceeds energy expenditure [42]. Important obesity-related dietary behaviors associated with a high energy intake are consuming energy-dense food (i.e. high in fat, salt, and sugars), drinking sugared drinks and alcohol, and consuming large portions of foods [42]. Energy can be expended by means of physical activity, which can be divided in different sub-behaviors such as active transportation (e.g. cycling to work), daily activities (e.g. cleaning), leisure time activities (e.g. walking), and sports [9]. Hence, physical activity and dietary intake are important targets in the prevention of obesity [9]. By improving these two behaviors the energy balance can be restored, which in turn will prevent weight gain. In line with the fact that individuals with low levels of education have the highest rate of obesity, they also tend to have the highest energy intake [43] and the lowest physical activity levels [44]. This confirms the importance of developing interventions that are appealing and effective for these individuals.

Prevention of obesity is thought to be most successful when focusing on small changes in dietary intake and physical activity, since small changes are easier to initiate and maintain than large changes as strict diets or following an intensive sport schedule [45]. Further, achieving small changes could lead to an increased self-efficacy, which in turn could stimulate people to make additional small changes [45]. This small changes approach assumes that changes of about 100 kilocalories (kcal) per day are sufficient to ensure an energy balance which is in equilibrium and accordingly prevent weight gain [45]. This is equal to about 2,000 more steps of walking per day or 20 more minutes of other physical activities per day. To consume 100 kcal less per day, individuals only have to consume a little less of energy-dense food or substitute food such as a regular soda for a diet soda or a snack for a piece of fruit. These kinds of moderate energy reductions are sufficient to result in sustained decreases in energy intake without increased feelings of hunger [46]. To lose a little weight (about five kilogram in one year), individuals have to make daily changes of 200 kcal.

Based on the small changes approach, the primary objective of the two web-based computer-tailored interventions is to realize small changes in physical activity and dietary intake of 100 or 200 kcal per day in order to prevent obesity (i.e. maintain weight or achieve modest weight loss) among Dutch adults with a healthy weight or limited overweight.

### Step 2: program objectives

The goal of step two is to formulate change objectives, which are the most proximal goals of an intervention that help in achieving the behavioral and weight goals. The two components that are needed to formulate change objectives are performance objectives (i.e. sub-behaviors within the desired behavior) and their determinants [40].

### Performance objectives

It has been suggested that in particular self-regulation concepts are important to successfully achieve weight management goals, such as identification of a problem, setting goals, making plans, monitoring weight and behavior, and having coping skills [39,47]. Evidence has, for example, shown that individuals with adequate self-regulation skills are more successful in changing complex and habitual behaviors such as physical activity and dietary intake as well as in achieving weight maintenance [48,49]. The performance objectives (PO’s) and accordingly the content of the interventions were, therefore, based on the concepts and phases of self-regulation models. For example, the first PO is ‘individuals decide to prevent weight gain: maintain current weight or lose a little weight’. An overview of all PO’s can be found in Additional file 1 (Table S1). It should be noted that although these PO’s follow a sequence of action, it is possible to return to previous PO’s.

### Determinants

The next step was to identify the most relevant (i.e. important and changeable) determinants of each PO. For this purpose we carried out an extensive literature review on determinant studies. During this process, the I-Change Model [37] was used as framework to identify determinants for the pre-motivational, motivational, and post-motivational phase. This model is an integration of various behavior-oriented theories such as the Social Cognitive Theory, Health Belief Model, Theory of Planned Behavior, Transtheoretical Model, and Goal Setting theories. Based on a determinant analysis, the most relevant determinants that need to be targeted in the intervention are knowledge, awareness, risk perception, attitude, social influence, self-efficacy, intention, skills, and action planning (see Additional file 1: Table S1).

### Change objectives

Change objectives (CO’s) specify what individuals have to learn in order to accomplish PO’s [40]. CO’s are created by combining the PO’s and determinants. In total, we formulated around 150 CO’s. An example of a CO concerning PO 6 (i.e. make coping plans to deal with difficulties) and the determinant knowledge is ‘individuals can explain how to deal with encountered difficulties’. Additional file 1 (Table S1) provides a selection of the most important CO’s per PO and determinant.

### Step 3: methods and applications

In the third step, theoretical methods for changing each determinant were selected and translated to practical applications. Methods and applications were retrieved from literature concerning behavior change techniques and study protocols of previous studies [40,50,51]. Additional file 1 (Table S1) provides an overview of the most important theoretical methods and practical applications of the program. When applicable, this table also shows the differences in practical applications between the text-text and video-text intervention. The most important methods and applications are elucidated in more detail below. It should be noted that both interventions have exactly the same educational content in order to allow for a direct comparison between the text-text intervention and the video-text intervention. For the video-text intervention, particularly the practical applications related to PO 1, 2, 7, and 8 are converted into videos.

### Providing information

To increase an individual’s knowledge and skills, providing information is included as theoretical method. In line with the parameters of this method (e.g. information must be relevant) [40], the program mainly consists of basic information that individuals are required to have in order to change their behavior successfully. Individuals will, for example, receive information about the small changes approach (PO 2). To prevent that participants have to read something that they already know or are not interested in, they mostly have the possibility to choose which information they want to read. The program, for example, consists of optional in-depth information, such as the amount of kcal in various food products, which can be read by clicking on hyperlinks. Important information, such as information about the small changes approach, is included in the regular sequence of the program, as this information is important for making further steps in the process. In both the video-text as text-text intervention, all information is provided by means of text-based messages.

### Consciousness raising and feedback

Consciousness raising and feedback will be used to increase individuals’ awareness about their body weight (PO 1 and 8), physical activity level, and dietary intake (PO 2, 7, and 9). For this purpose, individuals’ have to answer various questions about, for example, their body weight and general dietary intake and physical activity pattern. Based on their answers, they will receive tailored feedback about how they can improve their weight and behavior. This feedback will help participants to set appropriate weight and behavior change goals. In the video-text intervention, all these messages will be delivered using videos. In these videos, the feedback is given by actors playing an expert in the field of nutrition and physical activity. Simultaneously with the video message, a table is shown below the videos consisting of very specific information, for example the different food products a participant consumes or a participant’s BMI. In the text-text intervention, the same table will be provided only accompanied with text-based messages.

### Decisional balance

To help participants decide which behavior change would suit them best (PO 2), a decisional balance format is included as well. For this purpose, individuals will be asked about their attitudinal, self-efficacy, and social influence beliefs regarding physical activity and dietary intake. Based on their answers, the program will suggest which behavior would be most suitable for them to change. A person who has indicated, for example, to have a positive attitude and sufficient self-efficacy expectations for a change in dietary intake will be advised to choose for a change in dietary behavior. Participants can decide whether or not they will work through this part of the program. This optional part will only be advised to individuals who indicate that they find it difficult to decide which behavior change would suit them best. Because this procedure is optional, this part is only elaborated by means of text and thus exactly the same in both versions of the intervention.

### Goal setting

Goal setting is used as a method to help participants attain the desired behavior change [52]. In the program, participants can select a goal from a list with pre-defined options (i.e. goals) for weight, diet, and physical activity. We used a closed-ended goal setting format to prevent the formation of inadequate goals. Participants will be instructed about how to select a goal by means of written text. To further help participants set appropriate goals, they will receive extensive feedback on their behavior and weight, as mentioned before. After this feedback, participants have to decide whether they want to maintain their current weight or lose a little weight (PO 1) and how they want to achieve their weight goal, either by increasing their level of physical activity, improving dietary intake or both (PO 2). The possibility to change both behaviors is included, because several studies have shown that multiple health behavior interventions result in greater weight loss [53], have higher participation rates [54], and may be more cost-effective [53]. Individuals subsequently have to choose specific sub-behavior(s) within the chosen main behavior. Within physical activity, individuals can choose from three sub-behaviors: active transportation, physical activity in leisure time, and sports. For dietary intake a distinction is made in: dairy produce, sandwiches, food at dinner, snacks, hot and cold beverages, and alcohol. Individuals can choose a maximum of two sub-behaviors per main behavior (i.e. dietary intake and physical activity). Finally, individuals also have to decide how they want to improve the chosen sub-behavior using again a pre-defined list with goals. For dietary intake, participants can choose to eat smaller portion sizes, consume a less energy-dense alternative or eat less of a certain food product. For example, an individual who chooses for snacks subsequently has to indicate whether he/she wants to eat fewer snacks, eat low energy-dense snacks or eat smaller portion sizes of snacks. For physical activity, individuals have to decide whether they want to increase the frequency or duration of activity. For example, an individual can decide to walk more times per week as well as to walk longer per time. As this whole goal setting application concerns choices and decisions individuals have to make, this part is exactly the same in both interventions and merely elaborated by means of text.

### Action, preparatory, and coping planning

Research has shown that intended behavior changes are more likely to result in action when behavioral intentions are translated into concrete plans [55,56]. Hence, after individuals have set goals, they have to form plans for action to achieve the intended goals (PO 3). Action plans are generally made by means of the ‘if-then’ format [56]. The ‘if’ specifies when a specific action has to be performed, while the ‘then’ describes what specific action will be taken. In the interventions, individuals have the opportunity to make three kinds of plans: action (i.e. plans to perform the specific behavior), preparatory (i.e. plans for preparation), and coping plans (i.e. plans to deal with difficult situations). Individuals first have to formulate one action plan for every chosen sub-behavior (e.g. if I have lunch break at work, then I will eat an apple instead of a candy bar). To realize the behavior change, individuals also have the opportunity to formulate a maximum of three preparatory plans for each chosen sub-behavior (e.g. If I go to the supermarket on Monday, I will buy five apples). Both the action and preparatory plans will be formulated using an open-ended ‘if-then’ format. This format was chosen since a small pilot study among members of the target population has shown that this was the most preferred method for making action and preparatory plans. The ‘if’ and ‘then’ are predefined and individuals have to complete this sentence by filling out their own ideas and preferences. Individuals will also receive examples of good plans to help them formulate appropriate plans.

Individuals often encounter barriers that can hinder a successful behavior change [37]. Hence, a coping planning element will be introduced in the program once participants have started with the behavior change. They will be given the opportunity to make coping plans for situations in which they experience or expect difficulties to successfully perform the intended behavior. For each sub-behavior, individuals can specify a maximum of three coping plans using a closed-ended format, as the pilot study has also shown that this format was preferred for coping plans. Individuals can choose difficult situations from a pre-defined list. This list is composed based on findings from previous studies [57,58] and a pilot study that was performed among members of the target population. To make a coping plan, individuals first have to choose which difficult situations they encountered or expect to encounter (e.g. a busy working day). Subsequently, they will receive feedback on how to deal with these situations followed by the opportunity to choose a coping response from a list for every chosen situation.

It should be noted that because of the large variety that can exist in plans and in order to provide clear examples of adequate plans, the planning part is carried out merely using textual messages. Hence, this part is exactly the same for the video-text and text-text intervention and there were no additional video messages.

### Modeling

To target individuals’ self-efficacy to carry out the desired behavior change and the corresponding plans (PO 6, 7, 8, and 10), modeling techniques are used. Narratives are used as a practical application for modeling. A narrative is a constructive format in which a sequence of events is described [59]. For each sub-behavior, two narratives are created. The narratives follow a chronological storyline in which a role model tells how his/her behavior change is going and how he/she deals with encountered difficulties. Each story consists of implicit tips and participants can choose whether they want to view these stories. In line with the parameters of modeling, all narratives show people who are struggling with their behavior change but eventually succeed in achieving their goal [40]. In the text-text intervention, the narratives merely consist of text, while the narratives in the video-text intervention are displayed by means of videos. In these videos an actor plays the role of a model (i.e. a person who also used the program). A different actor is used for each of the eighteen narratives in order to increase the credibility of the stories. To ensure that participants can identify themselves with the persons in the narratives, we used a variety of actors with differences in ethnic background, age, gender, educational level, and BMI. In the text-text intervention, these characteristics were described in text in the introduction of the narratives.

### Reinforcement and external attribution

Besides modeling, participants’ self-efficacy will also be targeted by means of reinforcement and external attribution. During the interventions, the progress toward enactment of plans and goal achievement of the participants will be evaluated. This evaluation will, for example, assess whether participants have gained, maintained, or lost weight (PO 7) as well as whether or not they have improved their physical activity level and/or dietary intake (PO 8). Participants who achieve their goals successfully will be complimented, while those who fail to achieve their goals will receive feedback in which the failure will be attributed to external factors that people cannot control in order to prevent a decrease in self-efficacy [60]. In the video-text intervention, this feedback will be provided by means of a video message, in which the progress of an individual is discussed. Simultaneously with the video message, a table is shown below the video consisting of an overview of participants’ changes in body weight and dietary intake and/or physical activity during the program. In the text-text intervention, the same table will be provided, but then accompanied with text-based messages.

### Step 4: program development

### Scope and sequence

**Figure 1:**
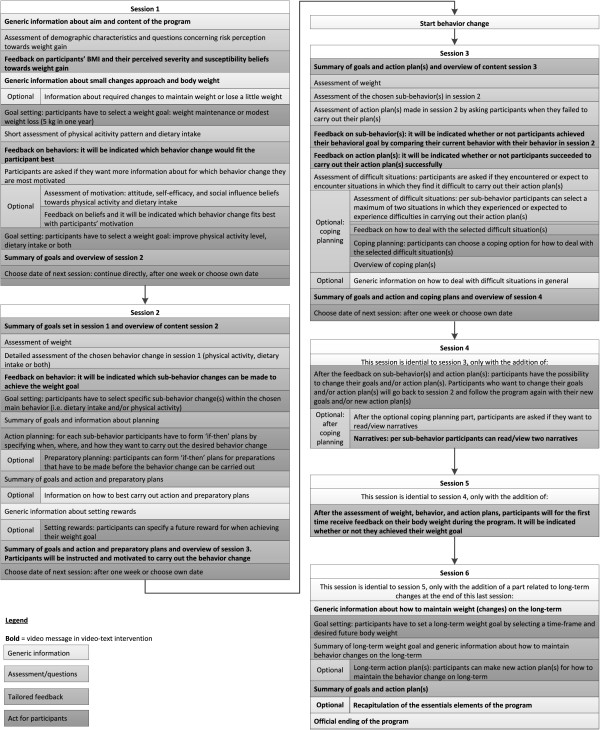
Overview of the content of the intervention.

The program consists of six sessions. Six sessions was considered to be sufficient to go through all phases of the I-Change Model and self-regulation theories. Figure 1 provides a flowchart of the six sessions, including a brief description of their content. In session one, participants have to decide whether they want to maintain their current weight or lose a little weight and how they want to achieve this goal: improving their physical activity level, dietary intake or both (PO 1 and 2). In session two, participants have to decide which specific sub-behaviors they want to change and how (PO 2), followed by the formation of action and preparatory plans (PO 3). It should be noted that the content of session one and two could have been combined into one large session, but because too much feedback may become too extensive for people to process [23], it was decided to use two separate sessions. However, at the end of session one, participants can decide to continue with session two directly. After session two, participants can start with enacting their plans and performing the desired behavior (PO 4 and 5). The remaining four sessions can be accessed in the next weeks, with at least one week between the sessions. The date for visiting the next sessions can be chosen by the participant. The focus of these sessions is on evaluating the progress toward meeting the weight and behavior change goals and enactment of the action plans and helping participants to deal with encountered or expected difficulties, for which they can make coping plans (PO 6, 7, and 8). In session four and five, participants have the possibility to change their goals and/or action plans (PO 9) and correspondingly go back to session two. In session four, five, and six, participants can further choose whether they want to view the narratives, which can help them in generating ideas for coping plans. Finally, the sixth session gives additional attention to how to maintain the behavior change on the long-term (PO 10). In total, participants can use the assigned intervention for a period of maximum three months, which is sufficient to follow all sessions. Each session lasts approximately 15 minutes, even though session two (in which current behavior is assessed) may take a bit longer to complete.

### Development

The two interventions are developed using the Tailorbuilder software (OSE, the Netherlands), a program which is specifically designed to develop web-based computer-tailored interventions. To enhance participant retention and outcomes [61], the interventions are integrated into a website (http://www.gewicht-in-balans.info). This website provides information about the study, the intervention, and answers to frequently asked questions. Participants have to log in to the website in order to use their assigned intervention. Once logged in, participants also have the opportunity to read information and advices from previous sessions.

For the video-text intervention 240 videos were recorded. Because of budgetary constraints it was unfortunately not possible to convert all educational messages into videos. In all video messages an actor reads the messages aloud (i.e. the text in the text-text intervention). The video messages related to physical activity were recorded in a gym and given by a professional male actor, while the messages related to dietary intake were recorded in a cooking studio using a professional female actor. These two actors had the role as an expert in the field of nutrition and physical activity. For the narratives, two actors per sub-behavior were recruited, who each had to act in three videos. These videos were recorded in a film studio that was decorated as a living room in order to increase the authenticity of the stories.

An example of the text-text intervention and the video-text intervention can be found in Additional file 2 and Additional file 3.

### Pretest of the interventions

Two pretests were conducted before a definite version of the interventions was developed. First, a prototype of the text-text intervention was pretested among 25 members of the target population. Twenty individuals worked through the intervention at home, while five participants participated in a cognitive interview in the presence of a member of the project team. During this interview, participants were asked to talk out loud about the choices they made in the program and about their interpretation of the feedback, usability of the action planning tools and so on. This process yielded useful findings, for example about log in problems and unclear questions and feedback. Based on these findings, the text-text intervention was improved after which the videos were recorded. Subsequently, a pretest of the video-text intervention was conducted using the same procedure as the first pretest. This second pretest, however, focused more on the clarity of the video messages as well as the usability of the website in which the interventions are integrated. This pretest again resulted in several useful suggestions for improvement, such as speed problems during the program and difficulties with navigation through the website.

### Step 5: planning for program implementation

The purpose of step five is to develop a plan for program use, including adoption, implementation, and continuation. The need to facilitate program use was, however, already taken into account from the beginning of step one by developing the interventions in such a way that will enhance their potential for being adopted, implemented, and sustained. For example, to implement the interventions no human actions are required. Individuals who want to use the intervention can go the website and register, after which they will automatically receive programmed e-mails. Furthermore, the development process of the interventions may also increase the likelihood of program use. For example, several small pilot studies among members of the target population were conducted to identify the preferences and needs of the potential users regarding the content of the intervention, the visual aspects of the website and intervention, the format of making preparatory, action, and coping plans, the recruitment materials for the evaluation study (e.g. brochure), the included difficult situations, the name of the intervention, and the actors in the video messages. During these pilot studies, we particularly investigated the opinions of individuals with a low level of education to ensure their preferences are integrated in the intervention and all intervention parts were clear to them. Moreover, by pretesting the text-text intervention and subsequently the video-text intervention, we also tried to adapt the intervention as much as possible to users’ preferences and needs.

To further facilitate program use, we also composed an advisory board consisting of potential adopters and implementers, including several occupational health services. Occupational health services have the objective to provide both cure and care for working people, which is in line with the purpose of the intervention. In the Netherlands, many employees receive an annual medical screening carried out by an occupational health service [62]. During these medical screenings, employees can be handed over a brochure about the intervention. Other implementation possibilities could be the use of advertisements in mass media, social media, and newsletters in companies.

### Step 6: planning for program evaluation

### Evaluation design and procedure

A three-group randomized controlled trial will be conducted to evaluate the efficacy of both versions of the intervention in comparison to a waiting list control group on BMI, dietary intake, and physical activity. Participants who will be randomized to the control group can use one of the interventions after the study. Measurements will take place at baseline (T0) and at 6 (T2) and 12 months (T3) after baseline. During all measurements, participants will have to complete an online questionnaire via the study website.

A power calculation was carried out to estimate the required number of participants. In order to detect a medium sized effect (d = 0.5) on BMI [63], using a power of .90, a significance level of .05, and being able to test interaction effects between participants with a low, medium, and high level of education, about 1,000 participants need to complete the study. Taking into account a drop-out percentage of 50% between baseline and the last follow-up measurement [64], this implies that approximately 2,000 participants are needed at baseline. These will be divided over the three study arms equally. Participants are eligible for inclusion when they are at least 18 years old, have a job, and have a BMI between 18.5 and 30. Although obese people (BMI ≥ 30) do not belong to the target population for the evaluation study, these individuals will be given the possibility to use the program. Participants who have a physical condition that may influence dietary or physical activity patterns (e.g. diabetes) are ineligible to participate. Participants will be recruited by various occupational health services during medical screenings as well as through companies without interference of the occupational health services. Brochures and messages in internal company newsletters will be used to invite people to participate in the study. Mass media advertisements and press releases will also be used to recruit participants. After registration at the study website, participants will be randomized to one of the three conditions and have the opportunity to fill out the baseline questionnaire. Participants in one of the intervention conditions have to wait two weeks before they can start using their assigned intervention. To decrease the likelihood of attrition in the evaluation study, one hundred cash prizes of €100 will be raffled among participants who complete all questionnaires [54]. Participants will also receive two reminders per intervention session and per measurement in order to improve retention in the study.

The Ethical Commission of the Open University Heerlen reviewed the study protocol and judged that the study is not within the scope of the Social Support Act (WMO) and that no approval of the medical ethics committee was required. They also indicated that there was no objection to performance of the study. The study is registered in the Dutch Trial Registry (nr. NTR3501). Participants’ approval will be obtained in line with the APA informed consent ethical principles. At the beginning of the study, all eligible participants will be provided with information about the study and asked to sign an online informed consent form.

### Hypotheses

The following hypotheses will be tested:

– The video-text intervention will be more effective than the text-text intervention in the prevention of weight gain (i.e. achieving weight maintenance or modest weight loss) and more appealing for individuals with low levels of education.

– The text-text intervention will be more effective than the video-text intervention in the prevention of weight gain and more appealing for individuals with high levels of education.

– Both the video-text as the text-text intervention will be more effective in the prevention of weight gain compared to the control group.

### Measurement instruments

The primary outcomes of the study are self-reported body weight, BMI, physical activity, and dietary intake. These will be assessed at baseline and at 6 and 12 month follow-up. In a sample of the participants, body weight and height will also be measured objectively by various occupational health services at baseline and 12 month follow-up.

Further, several potential mediators and moderators of the intervention effects will be measured. Mediators (i.e. working mechanisms of the interventions) that will be assessed include self-regulation skills and self-efficacy and intention towards being more physically active and eating less of energy-dense food products. These will be measured at baseline and at 6 and 12 month follow-up. At baseline, various moderators will be measured as well, including demographic characteristics (e.g. gender, age, and educational level), information processing style, self-efficacy, intention, media preference, and perceived body image.

**Figure 2:**
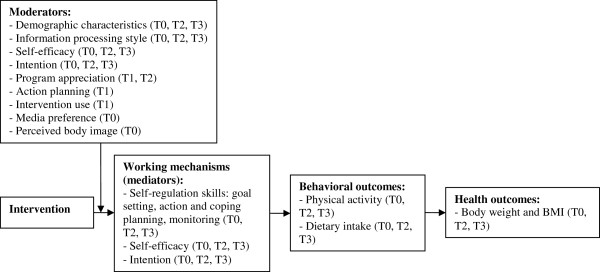
**Overview of concepts that will be measured.** Measurement times: T0 = baseline; T1 = after intervention session 6; T2 = 6 month follow-up; T3 = 12 month follow-up.

In addition, we will also conduct a process evaluation to examine potential moderating factors. This evaluation will take place directly after the last session of the intervention (T1) as well as briefly at the 6 month follow-up measurement (T2). Process measures that will be examined include action planning (i.e. the quality of action plans), intervention use (e.g. number of times participants logged in and amount of information viewed), and participants’ appreciation of the intervention (e.g. source, message, and channel factors). Figure 2 gives an overview of the concepts that will be measured at each time point.

## Discussion

The aim of the present paper was to describe the systematic development of a text-driven and a video-driven web-based computer-tailored intervention aimed to prevent obesity among lower and higher educated Dutch adults with a healthy weight or limited overweight. Both interventions have exactly the same educational content but differ in the format in which the information is delivered. The video-text intervention provides the core messages by means of videos, while the text-text intervention is fully text-based without the use of visual elements. Both interventions follow the steps distinguished in the I-Change Model and self-regulation theories; individuals will be made aware of their health risk behavior, motivated to improve their dietary or physical activity behavior, set goals, make plans, monitor and evaluate their weight and behavior, and deal with difficult situations in order to achieve weight maintenance or modest weight loss.

The video-text intervention is specifically developed to respond to the need to develop interventions aimed at individuals with a low level of education. Despite the fact that these individuals have the highest rate of obesity [5,6] and unhealthy dietary and physical activity patterns [43,44], there is a lack of interventions that are appealing and effective for this high risk group. The video-text intervention may be a good way to reach these individuals, as research has shown that lower educated individuals have a preference for videos over text and evaluate video messages as more attractive and understandable than text [26,30-32]. A related advantage of the video-text intervention is the fact that videos may reduce the amount of cognition needed to process information, which will free resources to process the main message [29]. By evaluating the interventions, insight will be obtained into whether the video-text intervention is indeed more effective and appealing for lower educated individuals than the text-text intervention.

Components that may increase the efficacy of both interventions in general are the self-regulation framework and the small changes approach. Evidence indicates that these are promising ways to prevent obesity [47-49]. The fact that we used a planned approach to develop the interventions (i.e. the IM protocol) may also increase the overall efficacy [39]. Moreover, by adapting the interventions to potential users as much as possible, by integrating their preferences as indicated in several small pilot studies, the efficacy of the interventions may also be facilitated. Additionally, the interventions offer a lot of freedom. Participants can, for example, choose which goals they want to achieve and which information and advices they do or do not want to read. It has been assumed that this high sense of autonomy will enhance individuals’ motivation, which in turn may increase the efficacy of the interventions [65].

To conclude, the use of the IM protocol has led to the development of two web-based computer-tailored interventions aimed to prevent obesity among Dutch adults with a healthy weight or limited overweight. By evaluating the interventions more insight will be obtained about their efficacy to prevent obesity, the efficacy of videos in web-based computer-tailored interventions, for which target group video tailoring is more effective, and by which processes effects may occur. These results can be used as input for future web-based computer-tailored interventions, specifically for those aimed at obesity prevention. If the interventions prove to be effective, an efficient obesity prevention tool that can reach large numbers of people against low costs will be available.

## Competing interests

HdV is the scientific director of Vision2Health, a company that licenses evidence-based innovative computer-tailored health communication tools. The other authors declare that they have no competing interests.

## Authors’ contributions

HdV and LL designed and wrote the original proposal. MJLW, AO, KS, LL, and HdV developed the obesity prevention program and execute the studies. MJLW significantly contributed to writing this paper, while AO, KS, LL, and HdV were involved in revising the manuscript critically. All authors read and approved the final manuscript.

## Pre-publication history

The pre-publication history for this paper can be accessed here:

http://www.biomedcentral.com/1471-2458/13/978/prepub

## Supplementary Material

Additional file 1: Table S1 Overview of performance objectives, determinants, change objectives, theoretical methods, and practical applications. Click here for file

Additional file 2 Example of the text-text intervention. Click here for file

Additional file 3 Example of the video-text intervention. Click here for file
