# Supplementary material for: Systematic development of a text-driven and a video-driven web-based computer-tailored obesity prevention intervention
Source: BMC Public Health. 2013 Oct 20;13:978. doi: 10.1186/1471-2458-13-978 (PMC4015713; doi:10.1186/1471-2458-13-978)
Supplement: Additional file 1: Table S1 — Overview of performance objectives, determinants, change objectives, theoretical methods, and practical applications. [file 1471-2458-13-978-S1.pdf]

Additional file 1. Table 1. Overview of performance objectives, determinants, change objectives, theoretical methods, and practical applications.

Performance objectives	Determinants	Change objectives	Theoretical methods	Practical applications
1. Individuals decide to prevent weight gain: maintain current weight (100 kcal) or lose a little weight (200 kcal)	Knowledge	Can explain what the energy balance is and which efforts are required to maintain weight and lose a little weight	Providing information	Video-text intervention: an actor (i.e. expert in the field of nutrition and physical activity) will give factual information about the energy balance and body weight. Participants will also receive basic information about the efforts that are required to maintain weight and lose a little weight.<br>Text-text intervention: this information is described in text.
	Awareness	Can express whether own body weight is healthy or not	Consciousness raising<br>Feedback	Video-text intervention: an actor will give feedback on participants' BMI by comparing their BMI to the BMI categories. Below the video message, participants' actual BMI will be shown.<br>Text-text intervention: this feedback is described in text.
	Risk perception	Acknowledge health consequences of weight gain and obesity	Personalize risk<br>Providing information	Video-text intervention: an actor will give feedback on participants' risk perception by giving information about participants' personal chances to gain weight in future as well as the consequences of being overweight and obese.<br>Text-text intervention: this feedback is described in text.
	Self-efficacy	Can indicate for which weight goal they have the highest level of confidence	Persuasion	Video-text intervention: an actor will give information about the size of the changes participants should make for maintaining weight or losing a little weight.<br>Text-text intervention: this information is described in text.
2. Individuals decide to make at least one small change in dietary intake and/or physical activity	Knowledge	Can explain which small changes are required in dietary intake and/or physical activity	Providing information	Video-text intervention: an actor will give factual information about the small changes approach as well as the required change in dietary intake and physical activity in order to maintain weight and lose a little weight.<br>Text-text intervention: this information is described in text.
	Awareness	Can express which behavior change (physical activity or dietary intake) offers most possibilities for improvement	Consciousness raising<br>Feedback	Video-text intervention: participants will fill out a questionnaire about their physical activity pattern and dietary intake. Based on their answers, an actor will give feedback on their behaviors and indicate which behavior change would be best for them.<br>Text-text intervention: this feedback is described in text.
	Attitude	Can indicate which behavior change they feel most positive about	Feedback<br>Decisional balance<br>Counter conditioning	Participants can choose advantages and disadvantages of dietary and physical activity behavior. Advantages will be confirmed, while disadvantages will be countered. It will also be indicated which behavior change they are most positive about.

	Self-efficacy	Can indicate for which behavior change they have the highest level of confidence	Feedback<br>Decisional balance	Participants will answer self-efficacy questions and based on their answers, it will be indicated which behavior change they are most confident about.
	Social influence	Can indicate for which behavior change they will receive most social support	Feedback<br>Information about others' approval<br>Decisional balance	Participants will answer social influence questions and based on their answers, it will be indicated for which behavior change they will get most support. They will also receive information about the use of social support.
3. Individuals make plans (action and preparatory) for how to carry out the behavior change	Knowledge	Can explain how to make plans	Providing information	Participants will receive information about the structure of good plans and how they can identify the most personal relevant 'if' and 'then'.
	Self-efficacy	Are able to make plans where they are confident about	Guided practice<br>Persuasion	Participants will be shown examples of good plans and informed how they can make plans step by step.
	Skills	Apply information to make plans	Implementation intentions<br>Providing information	Based on the 'if-then' format, participants will make plans for how to carry out the behavior change.
4. Individuals start with the chosen behavior change	Self-efficacy	Express confidence in ability to perform desired behavior	Persuasion	The plans and goals will be summarized and participants will be explained that by using their plans they are capable of performing the desired behavior.
	Skills	Are able to perform the desired behavior	Providing information	Video-text intervention: an actor will give information about how to apply action and preparatory plans in practice.<br>Text-text intervention: this information is described in text.
5. Individuals monitor their behavior and weight	Skills	Are able to monitor behavior change and compare it with goal	Self-reevaluation	Video-text intervention: an actor will instruct participants to compare their behavior with the desired behavior as specified in their plans.<br>Text-text intervention: this information is described in text.
	Self-efficacy	Have confidence to deal with deviations from the desired behavior	Persuasion	Video-text intervention: an actor will inform participants how they can monitor their behavior and how to take action when the desired behavior is not performed adequately.<br>Text-text intervention: this information is described in text.
6. Individuals make coping plans how to deal with difficulties during the behavior change	Knowledge	Can explain how to deal with difficult situations	Providing information	Participants will receive information about how to deal with expected or encountered difficult situations during the behavior change.
	Self-efficacy	Are able to make coping plans where they are confident about	Guided practice<br>Modeling	Participants will be shown examples of good coping plans and informed how they can make coping plans step by step. They will also receive examples of how other people (i.e. actors) deal with difficult situations during the behavior change (narratives).
	Skills	Apply information to make coping plans	Implementation intentions<br>Providing information	Based on the 'if-then' format, participants will make coping plans for how to deal with difficult situations.

7. Individuals evaluate their behavior change	Awareness	Recognize when behavior change is (un)successful	Consciousness raising<br>Feedback	Video-text intervention: an actor will indicate whether or not participants have achieved their behavior change goal by comparing participants' current behavior with their behavior at the start of the program. Below this video message, a table will be shown that provides an overview of participants' current and previous behavior.<br>Text-text intervention: this feedback is described in text and includes the same table.
	Self-efficacy	Have confidence about how to evaluate behavior change	Reinforcement<br>External attribution<br>Modeling	Video-text intervention: an actor will reinforce participants who achieved their behavior change goal. Failure will be attributed to external factors. Multiple actors will further also act in role model stories (narratives) by serving as examples of how other people deal with (un)successful behavior changes.<br>Text-text intervention: this feedback and the narratives are described in text.
8. Individuals evaluate their weight change	Awareness	Recognize when weight change is (un)successful	Consciousness raising<br>Feedback	Video-text intervention: an actor will indicate whether or not participants achieved their weight goal by comparing participants' current weight with their weight at the start of the program. Below this video message, a table will be shown that provides an overview of participants' weight during the program.<br>Text-text intervention: this feedback is described in text and includes the same table.
	Self-efficacy	Have confidence about how to evaluate weight change	Reinforcement<br>External attribution<br>Modeling	Video-text intervention: an actor will reinforce participants who have achieved their weight goal. Failure will be attributed to external factors. Multiple actors will further also act in role model stories (narratives) by serving as examples of how other people deal with (un)successful weight changes.<br>Text-text intervention: this feedback and the narratives are described in text.
9. Individuals decide to maintain the behavior change or to make a new behavior change (and go back to PO 2)	Awareness	Are aware of their weight and behavior change progress	Consciousness raising<br>Feedback	Video-text intervention: an actor will indicate whether or not participants achieved their weight and behavior change goals.<br>Text-text intervention: this information is described in text.
	Self-efficacy	Have confidence in weight and behavior change goals	Persuasion<br>Reinforcement<br>External attribution	Video-text intervention: an actor will indicate that participants who achieved their weight goal successfully can try to make a more challenging behavior change. Participants who failed to achieve their weight goal will be asked if they want to make an easier behavior change.<br>Text-text intervention: this information is described in text.

10. Individuals maintain the behavior change	Knowledge	Can tell how to maintain behavior change on long-term	Providing information	Video-text intervention: an actor will give information about how participants can maintain behavior changes on the long-term.<br>Text-text intervention: this information is described in text.
	Skills	Apply information to set a long-term weight goal	Providing information	Participants will set a long-term weight goal by choosing a time period and a desired future weight.
	Self-efficacy	Have confidence to maintain behavior change	Providing information<br>Persuasion	Video-text intervention: an actor will explain that participants can maintain behavior change(s) by means of the skills they have acquired during the intervention.<br>Text-text intervention: this information is described in text.
	Intention	Are motivated to maintain behavior change	Arguments	Video-text intervention: an actor will give factual information about the advantages of maintaining behavior changes on the long-term.<br>Text-text intervention: this information is described in text.
