# Supplementary figures and images for: Systematic development of a text-driven and a video-driven web-based computer-tailored obesity prevention intervention
Source: BMC Public Health. 2013 Oct 20;13:978. doi: 10.1186/1471-2458-13-978 (PMC4015713; doi:10.1186/1471-2458-13-978)

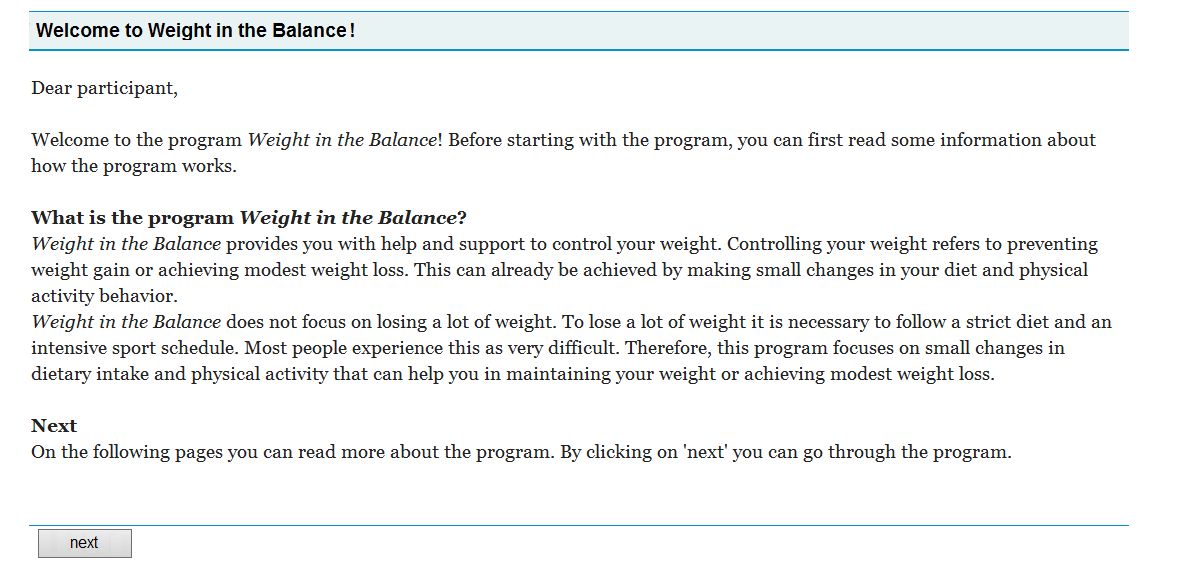

Supplement: Additional file 2 — Example of the text-text intervention. [file 1471-2458-13-978-S2.png]

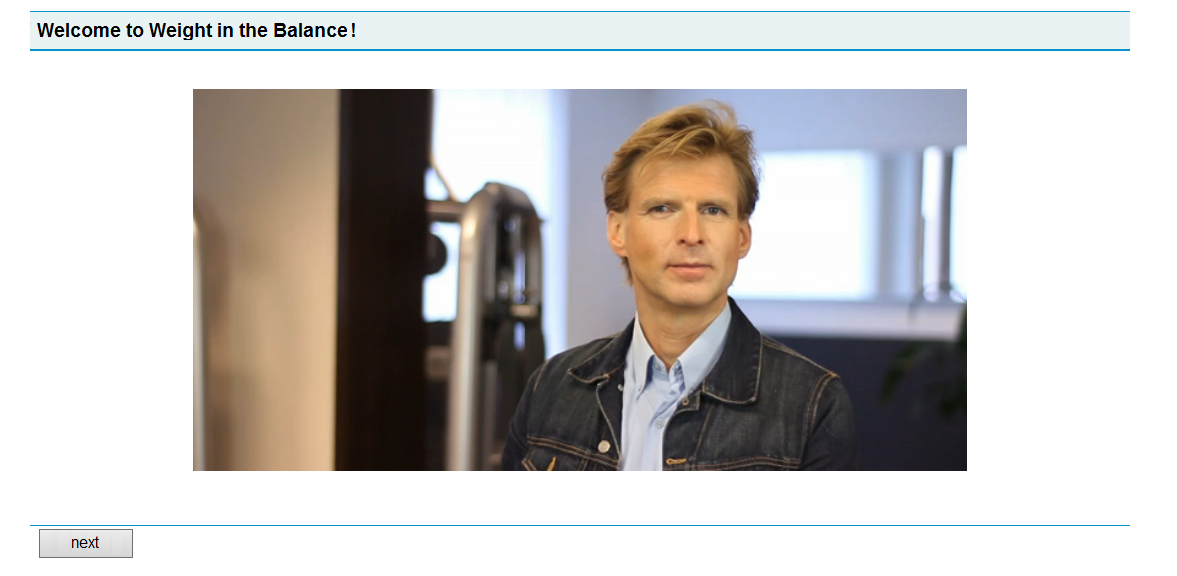

Supplement: Additional file 3 — Example of the video-text intervention. [file 1471-2458-13-978-S3.png]
